# Novel bovine hepacivirus in dairy cattle, China

**DOI:** 10.1038/s41426-018-0055-8

**Published:** 2018-04-04

**Authors:** Gang Lu, Kun Jia, Xiaokun Ping, Ji Huang, Aijian Luo, Peixin Wu, Shoujun Li

**Affiliations:** 1 College of Veterinary Medicine, South China Agricultural University, 510642 Guangzhou, Guangdong Province China; 2 Guangdong Provincial Key Laboratory of Prevention and Control for Severe Clinical Animal Diseases, 510642 Guangzhou, Guangdong Province China; 3 Guangdong Technological Engineering Research Center for Pet, 510642 Guangzhou, Guangdong Province China

Since it was first identified in 1989, Hepatitis C virus (HCV) has long been regarded as having one true natural host—humans^1^. However, after 2011, novel HCV-like viruses have been discovered in a wide range of hosts, including dogs, horses, rodents, bats, monkeys, cows, donkeys, and sharks^2–5^. Among them, bovine hepacivirus (BovHepV) was first reported in Ghana and Germany in 2015 by two separate research groups with a prevalence of 8.5% (9/106) and 1.6% (5/320), respectively^6, 7^. The BovHepV genome encompasses 9K–10K nt and has a 5′ UTR, a 3′ UTR, and a long open reading frame (ORF) of 8319 or 8322 nt encoding a single polyprotein. Genomic analysis based on the polyprotein gene demonstrated that BovHepV genetically diverged from other previously reported HCV-like viruses. In 2017, a novel genetic group of BovHepV was found in Brazilian cattles by detecting 6 serum samples, though only a 217 bp 5′ UTR fragment was sequenced^8^. These studies demonstrated that BovHepV has genetic diversity and has a distinct geographical pattern of genetic groups. Understanding the prevalence and genome information of BovHepV globally is important for assessing the global epidemiology and evolutionary pattern of this emerging virus.

Cattle have a large population size, and are important sources for both meat and milk production. Cattle have multiple ways to contact human and other animals. Before the risk of BovHepV on public health is studied, its potential to establish zoonotic infections should not be underestimated. In the present study, we identified and genetically characterized BovHepV in dairy cattle in China, which diverged from viruses found in other countries.

In March, 2017, a total of 102 serum samples were collected from dairy cattle in one farm in Guangdong Province, Southern China. The ages of these animals ranged from 1.1 to 11.8 years (Supplementary Table S1). Total RNA was extracted from 200 µL of serum from each sample using RNAiso Plus (Takara, Japan) following the manufacturer’s protocol, and was diluted in 20 μL nuclease-free water. cDNA was synthesized using 7 μL RNA, random primers, and GoScript reverse transcription system (Promega, USA). Screening for BovHepV infections was performed by using semi-nested PCR. The primers were designed on the basis of the conservative region observed in the published BovHepV 5′ UTR sequences (Supplementary Table S2). After two rounds of PCR and electrophoresis on a 1.5% agarose gel, a serum sample yielding a PCR product of ≈200 bp was considered to be positive for BovHepV. The PCR product with the expected size was purified using AxyPrep DNA gel extraction kit (Axygen, USA) and was then sent for Sanger sequencing from both ends (BGI, China). The viral load in the positive serum samples was determined using real-time PCR method. Briefly, a total of 8 µL viral RNA was reverse transcribed into cDNA using PrimeScript RT Master Mix (Takara, Japan) with random primers. Real-time PCR was performed under the following conditions: 95 °C for 5 min, followed by 45 cycles of 95 °C for 10 s, 60 °C for 10 s, and 72 °C for 20 s, and 1 cycle of 95 °C for 5 s and 65 °C for 1 min, using SYBR Green I Master Kit (Takara, Japan) and the primers BovHepV64F and BovHepV264R (Supplementary Table S2). The BovHepV RNA was in vitro-transcribed and was used to generate the standard curve.

The blast result in the NCBI database (https://www.ncbi.nlm.nih.gov/) indicated 8 field serum samples containing BovHepV RNA and that displayed a nucleotide homology of 86.9–95.3% with other BovHepV strains. The serum samples had a viral load ranging from 1.3 × 10^4^ to 6.2 × 10^6^ copies/ml detected by real-time PCR. However, no viral RNA was detected in the milk samples collected from the 102 cattle. In this study, two BovHepV strains (BovHepV/GD/01, BovHepV/GD/02) yielded a bright band detected by semi-nested PCR and had a high viral load (5.7 × 10^6^, 6.2 × 10^6^ copies/ml), and their genomes were sequenced. Briefly, all of the BovHepV genomes available in the NCBI database were retrieved and aligned by Bioedit 7.0.9.0. Using a long-range and primer walking strategy, three primer pairs targeting the BovHepV genomes were designed by Oligo 7.0 (Supplementary Table S2), and the genome of BovHepV/GD/01 and BovHepV/GD/02 was amplified using Q5 high-fidelity DNA polymerase (NEB, UK). The purified blunt-ended viral fragments were cloned into the PLB vector using the lethal based fast cloning kit (TianGen, China) and were then transformed into *TOP10* chemically competent cell (Weidi, China). After sequencing, the raw data was assembled by SeqMan 7.1.0 and the near-complete genome of both Chinese BovHepV strains was submitted to the GenBank database (accession number: MG257793, MG257794).

The genome of both BovHepV strains had a polyprotein gene of 8325 nucleotides with a similar G + C content of 52.3% to the strains identified in other countries (51.2–52.7%). A total of 43 nucleotide site mutations occurred between the two field strains. Three unique nucleotide inserts (^32^AAC^36^) were found in the Chinese BovHepV strains, and 21 nucleotide deletions (^6242^GCAAGTGGCCGAGTCCTACGC^6243^) were observed in both of the Chinese and Ghanaian BovHepV strains compared with the Germany strains. After performing multiple sequence alignments by the Cluster W method, the sequence identity of the BovHepV polyprotein gene was calculated and displayed using MegAlign 7.1.0. The identity of the nucleotide sequences of the polyprotein gene between BovHepV/GD/01 and BovHepV/GD/02 was 99.7%. The nucleotide identities between members of BovHepV in China and other countries ranged from 77.8% to 81.7%. Considering a reference in *Flavivirus* that a novel genotype of viral species is defined if its nucleotide sequence homology is less than 84%^9^, the BovHepV strains in China were classified as a novel genotype. The Germany BovHepV strains, two Ghanaian BovHepV strains (GHC52, GHC55), and the other three Ghanaian BovHepV strains (GHC100, GHC25, GHC85) were also noted to differ with a nucleotide homology of <84%, representing three other genotypes. These four genotypes were provisionally named China, Ghana-1, Ghana-2, and Germany genotypes, respectively. The genome sequence analysis in our study confirmed the high diversity within these BovHepV strains.

The nucleotide sequences of the polyprotein gene of BovHepV/GD/01 and BovHepV/GD/02, five Ghanaian BovHepV strains, and five Germany BovHepV strains were processed for phylogenetic analyses, which were performed using MrBayes 3.2.1 with the GTR + Gamma nucleotide substitution model, Markov chain, Monte Carlo algorithm run with 4 chains under a general time-reversible model with 100 million generations (average standard deviation of split frequencies: 0.005) (Fig. 1). It clearly revealed that BovHepV was grouped into two clades. One clade was composed of BovHepV of the Germany genotype, and the other clade included BovHepV of the Ghana-1, Ghana-2, and China genotypes. BovHepV/GD/01 and BovHepV/GD/02 were observed to cluster together. Interestingly, BovHepV of the China genotype had a closest relationship with the Ghana-2 genotype, and viruses of both genotypes shared the same ancestor with the Ghana-1 genotype. The prevalent field BovHepV strains in China may have been transmitted from Ghana or other African countries. However, this finding requires further studies.

**Fig. 1:**
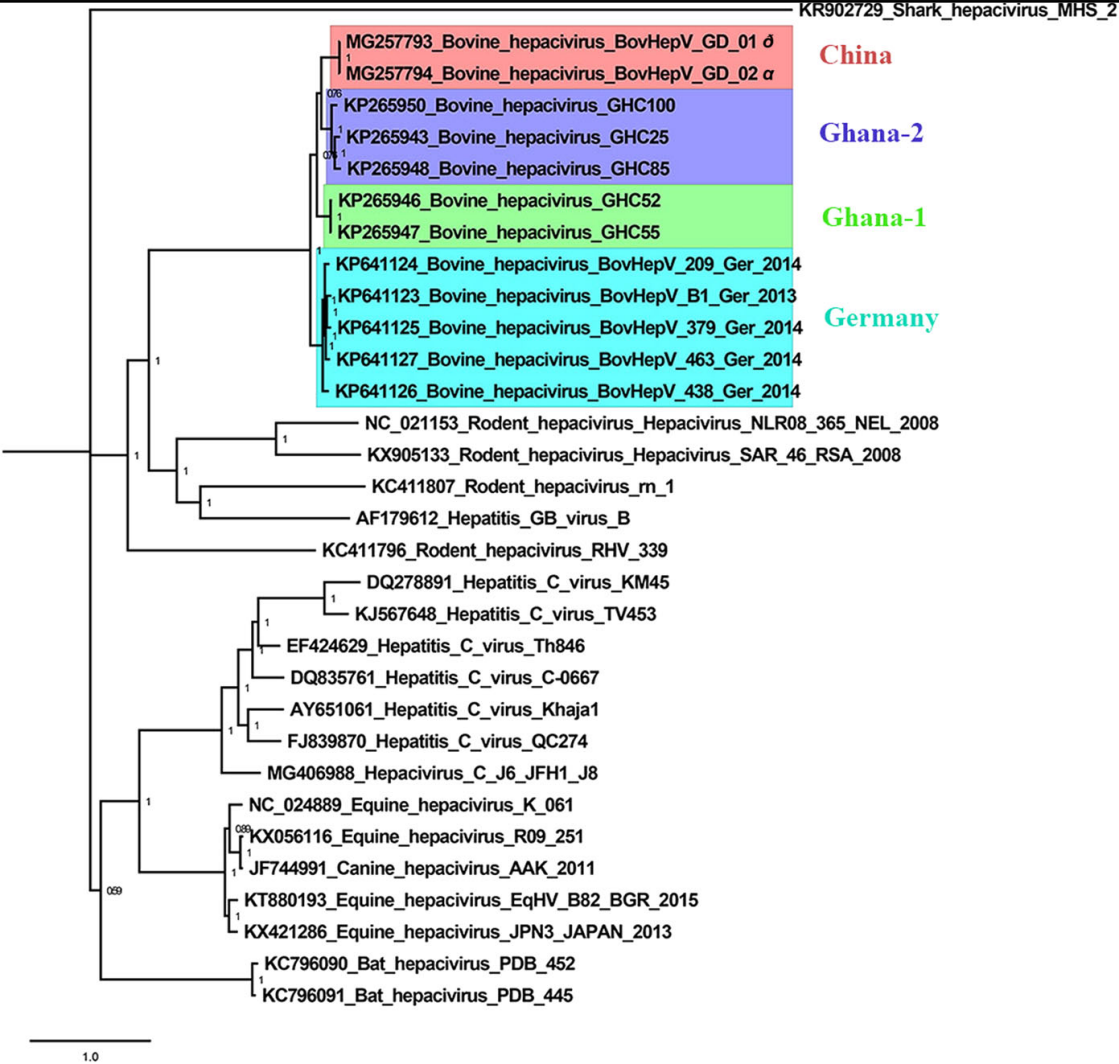
The phylogenetic tree constructed using the polyprotein gene of bovine hepacivirus strains identified in China, Germany, and Ghana, and the hepacivirus strains from human and other animals. Bayesian posterior probability values are shown in each clade to provide statistical support

In summary, we first identified BovHepV in dairy cattle in China and found through genome sequencing and analysis that it was a novel genotype. In addition, our study demonstrated that BovHepV genome had high genetic diversity. As a major human pathogen, HCV is a great threat to human health. To date, it is unclear whether the HCV-like virus of BovHepV can infect humans and affect human health. Further studies are necessary to understand the epidemiology and the evolutionary history of this virus, and its potential zoonotic transmission risk should be evaluated.

## Electronic supplementary material

Supplementary Table S2(DOCX 13 kb)

Supplementary Table S1(DOC 102 kb)

## References

[1] Choo QL (1989). Isolation of a cDNA clone derived from a blood-borne non-A, non-B viral hepatitis genome. Science.

[2] Scheel TK, Simmonds P, Kapoor A (2015). Surveying the global virome: identification and characterization of HCV-related animal hepaciviruses. Antivir. Res..

[3] Hartlage AS, Cullen JM, Kapoor A (2016). The strange, expanding world of animal hepaciviruses. Annu. Rev. Virol..

[4] Burbelo PD (2012). Serology-enabled discovery of genetically diverse hepaciviruses in a new host. J. Virol..

[5] Kapoor A (2011). Characterization of a canine homolog of hepatitis C virus. Proc. Natl Acad. Sci. USA..

[6] Corman VM (2015). Highly divergent hepaciviruses from African cattle. J. Virol..

[7] Baechlein C (2015). Identification of a novel hepacivirus in domestic cattle from Germany. J. Virol..

[8] Canal CW (2017). A novel genetic group of bovine hepacivirus in archival serum samples from brazilian cattle. Biomed. Res. Int..

[9] Kuno G, Chang GJ, Tsuchiya KR, Karabatsos N, Cropp CB (1998). Phylogeny of the genus Flavivirus. J. Virol..

