# Supplementary material for: Novel bovine hepacivirus in dairy cattle, China
Source: Emerg Microbes Infect. 2018 Apr 4;7:54. doi: 10.1038/s41426-018-0055-8 (PMC5883034; doi:10.1038/s41426-018-0055-8)
Supplement: Supplementary file 1 — Supplementary Table S2(DOCX 13 kb) [file 41426_2018_55_MOESM1_ESM.docx]

**Supplementary Table S2 Primers used to screen and sequence the bovine hepacivirus in this study**

| **Primer name** | **Primer sequence (5’-3’)** | **Length (bp)** |
| --- | --- | --- |
| BovHepV3F^a^ | ATCRACACTCCAGGCTCAYG | 262 |
| BovHepV64F^b^ | AGTAGGAGGCGCCTATCCC | 201 |
| BovHepV264R^a,b^ | TGCGGCAGGACCCTATCA |  |
| BovHepV 63F | TAGTAGGAGGCGCCTATCCC | 1520 |
| BovHepV1582R | TTNACCTTRTAYTCRCACCC |  |
| BovHepV1446F | TCCGTTGAGGACATGTGGTG | 3578 |
| BovHepV5023R | AGYTGYACCCKYTCDACCCA |  |
| BovHepV4920F | GACCCTGCTGAAGATGACGA | 3880 |
| BovHepV 8799R | CGGGTAGCCCCTTATTACGC |  |

Primers were numbered according to the sequence of BovHepV strain GHC25.

^a^ Primers used in the first round of semi-nested PCR

^b^ Primers used in the second round of semi-nested PCR and real-time PCR.

N: A, T, C, G; Y: C, T; R: A, G; D:G, A, T.
