# Supplementary material for: Novel bovine hepacivirus in dairy cattle, China
Source: Emerg Microbes Infect. 2018 Apr 4;7:54. doi: 10.1038/s41426-018-0055-8 (PMC5883034; doi:10.1038/s41426-018-0055-8)
Supplement: Supplementary file 2 — Supplementary Table S1(DOC 102 kb) [file 41426_2018_55_MOESM2_ESM.doc]

**Supplementary Table S1 Characteristics of the cattle in this study**

| Animal Number | Animal Age (Year) | Infection status^a^ | Viral load (copy number/mL) |
| --- | --- | --- | --- |
| 001 | 5.1 | Negative |  |
| 002 | 7.3 | Negative |  |
| 003 | 11.5 | Negative |  |
| 004 | 6.8 | Negative |  |
| 005 | 11.6 | Negative |  |
| 006 | 3.8 | Negative |  |
| 007 | 4.9 | Negative |  |
| 008 | 5.2 | Negative |  |
| 009 | 3.9 | Negative |  |
| 010 | 3.7 | Negative |  |
| 011 | 6.1 | Negative |  |
| 012 | 3.8 | Negative |  |
| 013 | 9.8 | Negative |  |
| 014 | 9.7 | Negative |  |
| 015 | 7.5 | Negative |  |
| 016 | 3.8 | Negative |  |
| 017 | 9.5 | Negative |  |
| 018 | 2.4 | Negative |  |
| 019 | 6.5 | Positive | 1.3×10^4^ |
| 020 | 5.8 | Negative |  |
| 021 | 7.9 | Positive | 1.6×10^5^ |
| 022 | 6.2 | Negative |  |
| 023 | 3.3 | Negative |  |
| 024 | 11.7 | Negative |  |
| 025 | 8.5 | Negative |  |
| 026 | 5.5 | Negative |  |
| 027 | 3.4 | Negative |  |
| 028 | 1.1 | Negative |  |
| 029 | 10.5 | Negative |  |
| 030 | 6.7 | Negative |  |
| 031 | 11.8 | Negative |  |
| 032 | 6.4 | Negative |  |
| 033 | 3.9 | Negative |  |
| 034 | 6.7 | Negative |  |
| 035 | 8.7 | Negative |  |
| 036 | 3.4 | Negative |  |
| 037 | 3.6 | Negative |  |
| 038 | 8.8 | Negative |  |
| 039 | 9.9 | Negative |  |
| 040 | 3.4 | Negative |  |
| 041 | 6.7 | Negative |  |
| 042 | 6.8 | Positive | 3.2×10^4^ |
| 043 | 7.9 | Negative |  |
| 044 | 7.7 | Negative |  |
| 045 | 5.4 | Negative |  |
| 046 | 8.6 | Negative |  |
| 047 | 3.3 | Negative |  |
| 048 | 2.8 | Negative |  |
| 049 | 3.7 | Negative |  |
| 050 | 3.4 | Negative |  |
| 051 | 2.5 | Negative |  |
| 052 | 3.8 | Negative |  |
| 053 | 4.8 | Negative |  |
| 054 | 3.7 | Positive | 4.7×10^4^ |
| 055 | 3.9 | Negative |  |
| 056 | 2.7 | Negative |  |
| 057 | 9.7 | Negative |  |
| 058 | 3.8 | Negative |  |
| 059 | 3.4 | Negative |  |
| 060 | 6.2 | Negative |  |
| 061 | 2.6 | Negative |  |
| 062 | 3.4 | Positive | 2.2×10^4^ |
| 063 | 3.7 | Positive | 2.1×10^4^ |
| 064 | 3.8 | Negative |  |
| 065 | 3.7 | Negative |  |
| 066 | 2.4 | Negative |  |
| 067 | 4.5 | Negative |  |
| 068 | 3.9 | Negative |  |
| 069 | 3.4 | Negative |  |
| 070 | 2.6 | Negative |  |
| 071 | 5.8 | Negative |  |
| 072 | 3.7 | Negative |  |
| 073 | 6.1 | Negative |  |
| 074 | 4.6 | Negative |  |
| 075 | 2.3 | Negative |  |
| 076 | 4.6 | Negative |  |
| 077 | 4.5 | Negative |  |
| 078 | 3.8 | Negative |  |
| 079 | 2.2 | Negative |  |
| 080 | 2.7 | Negative |  |
| 081 | 2.8 | Negative |  |
| 082 | 2.5 | Negative |  |
| 083 | 5.1 | Negative |  |
| 084 | 7.7 | Negative |  |
| 085 | 7.2 | Negative |  |
| 086 | 3.3 | Negative |  |
| 087 | 4.7 | Negative |  |
| 088 | 6.4 | Negative |  |
| 089 | 4.6 | Negative |  |
| 090 | 2.1 | Negative |  |
| 091^b^ | 3.1 | Positive | 5.7×10^6^ |
| 092 | 3.7 | Negative |  |
| 093 | 4.5 | Negative |  |
| 094 | 2.4 | Negative |  |
| 095^b^ | 3.4 | Positive | 6.2×10^6^ |
| 096 | 3.9 | Negative |  |
| 097 | 11.7 | Negative |  |
| 098 | 6.8 | Negative |  |
| 099 | 11.8 | Negative |  |
| 100 | 4.4 | Negative |  |
| 101 | 5.1 | Negative |  |
| 102 | 7.6 | Negative |  |

^a^The BovHepV viral RNA in the serum was detected by two rounds of PCR.

^b^The BovHepV genome in the two animals was sequenced in this study.
